# Application of Imbalanced Data Classification Quality Metrics as Weighting Methods of the Ensemble Data Stream Classification Algorithms

**DOI:** 10.3390/e22080849

**Published:** 2020-07-31

**Authors:** Weronika Wegier, Pawel Ksieniewicz

**Affiliations:** Department of Systems and Computer Networks, Wroclaw University of Science and Technology, 50-370 Wroclaw, Poland; 225935@student.pwr.edu.pl

**Keywords:** data streams, imbalanced data, classification, classifier ensembles, oversampling

## Abstract

In the era of a large number of tools and applications that constantly produce massive amounts of data, their processing and proper classification is becoming both increasingly hard and important. This task is hindered by changing the distribution of data over time, called the concept drift, and the emergence of a problem of disproportion between classes—such as in the detection of network attacks or fraud detection problems. In the following work, we propose methods to modify existing stream processing solutions—Accuracy Weighted Ensemble (AWE) and Accuracy Updated Ensemble (AUE), which have demonstrated their effectiveness in adapting to time-varying class distribution. The introduced changes are aimed at increasing their quality on binary classification of imbalanced data. The proposed modifications contain the inclusion of aggregate metrics, such as F1-score, G-mean and balanced accuracy score in calculation of the member classifiers weights, which affects their composition and final prediction. Moreover, the impact of data sampling on the algorithm’s effectiveness was also checked. Complex experiments were conducted to define the most promising modification type, as well as to compare proposed methods with existing solutions. Experimental evaluation shows an improvement in the quality of classification compared to the underlying algorithms and other solutions for processing imbalanced data streams.

## 1. Introduction

Data stream analysis has recently become an increasingly popular topic in the pattern recognition field [1,2]. A multitude of tools and applications constantly produces huge volumes of data that should—most often in a limited time—be processed to extract valuable information. Examples of such sources include, for example, social media and recommendation systems [3], or particularly, increased network traffic during the era of coronavirus and remote work [4]. Such data differ significantly from static data sets, introducing additional difficulties in constructing effective models to solve learning tasks. In addition, more and more often, for example, in the case of fraud detection [5] or network attacks [6], they introduce an imbalance problem [7,8], which is not negligible already when training on static data sets, making streaming classification even more challenging.

The problem of imbalanced data occurs when the size of one of the problem classes far exceeds the count of the other. It is not precisely determined by what numbers we may talk about imbalance, but it is often assumed [8] that in the 9:1 ratio we have a slight imbalance, and when it is 1000:1 or more, we are dealing with a very high imbalance.

Imbalanced data classification is a demanding task, because the dominant majority of recognition algorithms were designed with the assumption of proportional prior probability of classes. The assumption of most traditional recognition models is to minimize the prediction error, often ignoring the presence of disproportions in the class counts, which leads to the bias of the build model towards the majority class, thus significantly worsening the discriminatory abilities regarding the minority class. In addition, it is very important to carefully select the experimental protocol and the quality assessment metrics used [9], because the most commonly applied classification metrics, such as accuracy, do not take into account the disparities in the problem classes and thus incorrectly assess the quality of the model. One of the available choices is aggregated metrics, such as F1-score, G-mean or balanced accuracy score [10], which by taking into account the recognition quality for all the problem classes are much better suited to the problem of imbalanced data.

Data streams are ordered sequences of information, arriving at high speed [11]. They are also potentially infinite and may change over time. One of the most important phenomena distinguishing the classification of data streams and static data is the so-called concept drift. It consists of changing the distribution of classes in the set—the posterior probability or even the proportion between individual classes [12]. This significantly affects the quality of the prediction, because it often turns out that they were trained on outdated data. One possible taxonomy of this phenomenon is division into three types, due to the dynamics and characteristics of the changes. Sudden drift occurs when the posterior probability at t+1 is completely different from that at *t*. In the case of gradual drift, the change in the concept is slower, and the data from both concepts (before and after the change) are mixed up. The last type is the incremental drift, in which the first concept smoothly changes into the second, without mixing them together.

The characteristics of data streams leads to some indefeasible requirements for classifiers operating in their environment: fast data processing in which each object may be presented for training only once, low memory consumption, the possibility of prediction at any time and the ability to adapt to the changing distribution of problem classes [13].

Data for the model can be provided in two ways-online or in batches. In the first case, the objects are processed individually at the moment they arrive, while in the second case the data is grouped into chunks of the same size and processed together. Online learning allows faster detection of concept drifts [14], while learning in batches is easier to implement and more computationally efficient.

The problem of imbalanced streams can be even more difficult to solve than each of its struggles separately. Not all standard methods for solving the imbalanced data problem are feasible in a streaming environment. If the model is learned incrementally, most of the popular sampling algorithms cannot be used, and even the very determination of the imbalance ratio is not trivial [15]. In the case of a data chunk, it is easier to specify at least temporary proportions between classes, but depending on the size of the chunk and number of minority patterns, not all sampling will be equally effective. In addition, due to the characteristics of the streams and speeds at which the data arrives, the computational efficiency of algorithms should also be taken into account.

There are three main groups of methods for improving model performance over imbalanced data: methods at the data level, at the algorithm level, and hybrid methods that most often use an ensemble approach to classification. Data-level methods are based on adapting the training set by changing the number of samples to allow standard machine learning algorithms to train and classify correctly. The simplest and most popular approach is random sampling, where objects are duplicated (*over-*) or removed (*undersampling*) in a random manner. It may, however, lead to the removal of patterns potentially valuable for recognition or duplication of non-valuable samples (e.g., noise or outliers). More complex methods are, for example, the SMOTE (Synthetic Minority Over-sampling Technique) algorithm [16], creating new synthetic samples based on neighboring minority class objects, ADASYN, creating more synthetic samples near objects difficult to classify [17], or the NCL (Neighborhood Cleaning Rule) algorithm, removing majority class objects that affect the misclassification of the remaining samples [18]. The methods of preventing data imbalance at the algorithm level transform the machine learning model in such a way as to alleviate its bias in choosing the majority class. One such approach is methods interfering with the cost function of the model [19]. It is modified in such a way that it grants a greater cost of minority class object recognition error. The disadvantage of such methods is the difficulty of choosing the correct cost of errors in the case of real problems. Another algorithm-based method can be the one-class classifiers [20]. By building each classifier on only one class, we get rid of the problem of favoring other classes. However, choosing the right classifiers may be difficult for more complex problems.

For the case of data streams, there are several ways to classify them. The basic model from single classifiers is VFDT (Very Fast Decision Tree)—a decision tree using the Hoeffding boundary (Hoeffding bound) to create branches. Other examples are traditional incremental classifiers that have been adapted to the requirements of data streams, such as neural networks, Bayesian methods, and minimum-distance algorithms. Another approach is classifier ensembles that, thanks to their modularity, easily adapt to non-stationary data streams [21]. In batch learning, a new classifier is often created when new instances appear that may replace the weakest model in the pool. Examples of classifier assembly algorithms are AWE (Accuracy Weighted Ensemble) [22], AUE (Accuracy Updated Ensemble) [23,24] or WAE (Weighted Aging Ensemble) [13].

Several approaches have been proposed to solve the problem of imbalanced data streams. One of them is to expand the window with minority class data [25]. This is to reduce imbalance based on non-synthetic data (as opposed to artificially increasing the number). This solution, however, does not take into account the possibility of changing the distribution of minority class over time, and also violates the principle stating that one sample should be used once. Another method is used, for example, by the incremental OOB and UOB [15]. They are based on online bagging, where for each member classifier the samples obtained are duplicated according to the Poisson distribution, and sampling (*oversampling* in the case of OOB and *undersampling* at UOB) is done by controlling the λ parameter. The disadvantage of incremental learning, however, is the problem with determining the proportion of classes.

The aim of the following work is to propose the modification of popular ensemble models so that they employ the imbalanced classification metrics in the weighting of classifier members and compare them with existing data stream processing solutions. The created algorithms may achieve higher quality classification on imbalanced streams, and the proposed methods may slightly improve the currently used algorithms. The paper shows preliminary research of the topic, thus it will focus on the binary classification task.

## 2. Methods

### 2.1. Accuracy Weighted Ensemble

Accuracy Weighted Ensemble is an example of a batch processing classifier that processes data in the form of chunks. Each of the models entering the pool uses the same training procedure, but is built around a different data block.

A significant problem in processing data streams is recognizing the point in time when the data has become obsolete. The method of deleting the oldest objects is often used. However, this creates another problem of choosing the appropriate time window after which the data will be forgotten. In the case of too large window, objects from the previous concept are further used in the prediction of the new concept. On the other hand, if the window size is too small, the classifiers may have insufficient data for proper generalization, which may result in overfitting and poor quality of the model. For this reason, AWE does not use window mechanics, only the evaluation of stored data (in the form of classifiers trained on them) in relation to the current concept, and not the time spent in the pool.

It has been proven that an ensemble trained on *k* blocks in a manner where each model is built on a different block achieves better quality (less prediction error) than a single classifier learned on all *k* blocks. The condition for this is, however, the assumption that each member classifier has a weight assigned in accordance with its adaptation to the current data distribution. In the case of AWE, it is assessed by estimating the error made by each member on the latest block, which is considered to best reflect the current distribution of classes. In its basic version, the member weights are equal to the difference between the mean square error of each classifier and the estimated mean square error of the random classifier.

(1) $$w_{i}=MSE_{r}-MSE_{i},$$

where $MSE_{r}$ equals

(2) $$MSE_{r}=\sum_{c}p\left(c\right){\left(1-p\left(c\right)\right)}^{2},$$

for p(c) being the prior probability of class *c*.

$MSE_{i}$ is calculated as follows

(3) $$MSE_{i}=\frac{1}{|S_{n}|}\sum_{\left(x,c\right)\in S_{n}}{\left(1-f_{c}^{i}\left(x\right)\right)}^{2},$$

where $S_{n}$ is the latest data chunk in a form where *x* is a feature vector with label *c*, $|S_{n}|$ is the number of patterns building the chunk and $f_{c}^{i}$ states the posterior probability of *i*-th classifier assigning pattern *x* to class *c*.

Steps of the AWE algorithm in the form of pseudocode are presented in Algorithm 1.
**Algorithm 1** AWE pseudocode.**Input:** S: new data chunk K: size of the ensemble C: ensemble of K classifiers**Output:** C: ensemble of K classifiers with updated weights Train new classifier $C^{\prime }$ with S; Calculate weight of $C^{\prime }$ based on 1 using cross-validation on S; **for**
$C_{i}$ in C **do**  Calculate weight of $w_{i}$ based on 1 **end for** C ← K classifiers with highest weights from $C\cup C^{\prime }$; **return** C;

### 2.2. Accuracy Updated Ensemble

The second algorithm analyzed in the following work, Accuracy Updated Ensemble, is inspired by the AWE, but at the same time gets solved some disadvantages, which are the problem with the selection of the correct size of the chunk and the function of weight selection.

The first disadvantage is caused by the fact that each member classifier is trained only on one chunk of data, and then remains unchanged. If the chunk size is too small, the classifier will not have enough data to build a proper model. On the other hand, if it is too large, it may include data from different concepts. The solution proposed by AUE is to update models of classifiers stored in the pool, not just to change their weights according to changes in concept. Thanks to this, if the distribution of classes between chunks remains unchanged, classifiers well matched to it will improve their quality (as if they were trained on a larger number of samples from the beginning). As a result, it is possible to reduce the size of the chunk without a fear that this will cause a deterioration in the quality of individual members. Training occurs when the weight of the ensemble member is greater than the estimated weight of the random classifier.

The other disadvantage of AWE is its weighting function. By its definition and procedure (cutting off classifiers weaker than the random classifier) it may silence the entire ensemble and make it impossible to predict. AUE proposes the following weight function for *i*-th team member:

(4) $$w_{i}=\frac{1}{MSE_{i}+\epsilon }$$

$MSE_{i}$ is calculated according to Equation (3), and ϵ guarantees that dividing by 0 should never occur.

In addition to the introduced corrections, AUE retains all the advantages of AWE: assigning weights when a new chunk arrives, so classifiers modeled on the outdated concepts do not have a big impact on the result of the final prediction. As a result, AUE achieves better than AWE quality for streams with a stationary concept or streams including gradual drifts, and for sudden drifts, quality is at least the same.

Pseudocode of the AUE algorithm is presented in Algorithm 2.
**Algorithm 2** AUE pseudocode.**Input:** S: new data chunk K: size of the ensemble C: ensemble of K classifiers**Output:** C: ensemble of K updated classifiers with updated weights Train new classifier $C^{\prime }$ on S; Estimate the weight of $C^{\prime }$ based on 4 using cross-validation on S; **for**
$C_{i}\in C$
**do**  Calculate weight $w_{i}$ based on 4; **end for** C ← K classifiers with the highest weights from $C\cup C^{\prime }$; **for**
$C_{e}$ in C **do**  **if**
$w_{e}>\frac{1}{MSE_{r}}$ and $C_{e}\neq C^{\prime }$
**then**   update $C_{e}$ with S  **end if** **end for**

The presented algorithms are not adapted to the classification of imbalanced data. The main reasons are the methods of assigning weights to ensemble members. They not only affect the fusion of classifiers (mostly being conducted by weighted voting), but also their composition as classifiers with the lowest weights are removed. In addition, in AUE, only members with sufficiently high weights are trained. The mean square error on which the weights are based in both AWE and AUE, as well as typical accuracy score, is not suitable for assessing the quality of a classifier for imbalanced problems. Its low value, which translates into a high weight value, may come from a significant bias towards the majority class, which is best demonstrated by the case of the model that always gives the object the prediction for the majority class [26].

### 2.3. Proposed Changes in AUE and AWE Algorithms to Deal with Imbalanced Classification Problem

For the aforementioned reasons, this paper proposes the application of metrics much better at assessing the quality of algorithms aimed for binary classification of imbalanced data. The first of the selected metrics is the F1-score [27], which aggregates the simple metrics of sensitivity—determining the accuracy of the minority class classification, and precision—indicating the probability of its correct detection.

(5) $$F1-score=2*\frac{Precision*Sensitivity}{Precision+Sensitivity}$$

The subsequent selected metrics aggregate, using different approaches, the sensitivity and the specificity score, which in the binary case indicates the accuracy of recognizing the negative (majority) class. The first is G-mean [28]—the geometric mean of sensitivity and specificity (Equation (6)), and the last one is balanced accuracy score [26]—their arithmetic mean (Equation (7)). The advantage of both these metrics is that they consider both improving the minority class classification, but also avoiding deteriorating the majority class classification.

(6) $$G-mean=\sqrt{Sensitivity*Specificity}$$

(7) $$balanced\;accuracy\;score=\frac{Sensitivity+Specificity}{2}$$

In the proposed models, these metrics were used to calculate the weights of ensemble members, and in the case of the AUE model—to estimate the weight of a random classifier based on the prior probability of classes.

In addition, the conducted study verified the impact of data sampling on the quality of classification. Random over- and *undersampling* methods were chosen because of their simplicity and low computational complexity in stream processing. In addition, in the case of large imbalance leading to a small number of minority class objects, they give similar results to other popular sampling methods.

Pseudocodes of AWE and AUE with added proposed modifications are presented in Algorithms 3 and 4.
**Algorithm 3** Pseudocode of imbalanced metric-driven models based on AWE.**Input:** S: new data chunk C: ensemble of classifiers K: size of the ensemble**Output:** C: ensemble of classifiers with updated weights X ← sampled S Train new classifier $C^{\prime }$ on X; Estimate weight of $C^{\prime }$ with cross-validation on S based on (5), (6) or (7); **for**
$C_{i}$ in C **do**  Calculate weight $w_{i}$ of $C_{i}$ on S based on (5), (6) or (7); **end for** C ← K classifiers with the highest weights from $C\cup C^{\prime }$; **for**
$C_{i}$ in C **do**  $w_{i}\leftarrow \frac{w_{i}}{\sum_{C_{i}inC\cup C^{\prime }}w_{i}}$ **end for** **return** C;

**Algorithm 4** Pseudocode of imbalanced metric-driven models based on AUE.**Input:** S: new data chunk C: ensemble of classifiers K: size of the ensemble**Output:** C: ensemble of updated classifiers with updated weights X ← sampled S Train new classifier $C^{\prime }$ na X; Estimate weight of $C^{\prime }$ using cross-validation on S based on 5, 6 or 7; **for**
$C_{i}$ in C **do**  Calculate weight of $C_{i}$ on S based on 5, 6 or 7; **end for** Calculate weight $w_{R}$ of random classifier on S based on 5, 6 or 6 and a priori probabilities; **for**
$C_{i}$ in C **do**  **if**
$w_{i}>w_{R}$
**then**   Update $C_{i}$ with S;  **end if** **end for** C ← K classifiers with the highest weights from $C\cup C^{\prime }$; **for**
$C_{i}$ in C **do**  $w_{i}\leftarrow \frac{w_{i}}{\sum_{C_{i}inC\cup C^{\prime }}w_{i}}$ **end for** **return** C;

## 3. Experimental Set-Up

When testing the quality of the proposed algorithms, it was decided to use synthetic data streams. Although they do not show how the models would cope with real problems, artificially generated data allow for more accurate analysis due to, among others, the fixed location of the concept drifts and the possibility of any number of replications. The data was provided by the generator from the stream-learn module, employing the Madelon principle [29] of problem synthetization, being present also in the popular scikit-learn module, adding the ability to change data distribution over time and other properties known in the field of stream classification. Additionally, in order to make recognition more difficult, a fixed label noise was inducted to 1% of samples.

In order to thoroughly analyze the behavior of the models, streams with different imbalance levels were created, where the minority class accounts for, respectively, 5%, 10%, 20% and 30% of the entire data stream. For each proportion, five occurrences of different types of concept drift—sudden or gradual—were included in streams and evenly distributed over time. The data stream was delivered to the incremental models in the form of 100 chunks, each with 500 patterns. The stream consisted, like in many analyses of this field [15], of two informative features. Each stream type has been replicated five times, with different random states. Descriptions of generated stream types are shown in Table 1.

For each data stream, ensembles of 10 members were built, with the Hoeffding tree chosen as the base classifier. Combined models were created with each combination of parameters—(1) the base algorithm, (2) weighing method and (3) type of sampling, which gave 22 considered solutions, presented in Table 2. In addition, they were compared with the non-modified AWE and AUE algorithms, as well as with the WAE, OOB and UOB approaches.

The models were tested using the Test-Then-Train experimental protocol, in which the incoming chunk is used first to evaluate the model and then to train it. The metrics used in model construction, i.e., F1-score, G-mean and balanced accuracy score, were selected also for evaluation. After conducting the experiments, the Wilcoxon test [30] was carried out on the results for observation pairs with four degrees of freedom and a significance level of 0.05.

The experiments were carried out in the Python environment using the scikit-learn [31], stream-learn [32], imbalanced-learn [33] and scikit-multiflow [34] libraries and own implementations of modified AWE and AUE methods. The source code of used algorithms as well as experimental procedure is published in a public repository on GitHub (https://github.com/w4k2/imbalanced-stream-ensembles).

## 4. Experimental Evaluation

As it may be observed from Table 3, Table 4 and Table 5, larger differences between the results of individual models occur in the case of streams with a greater imbalance—both in terms of the average of all scores achieved during processing as well as in accordance with statistical tests. Only a large disproportion between classes, on the order of, for example, 1:19, 1:9, seems to be a proper challenge, significantly differentiating the quality of the presented algorithms.

As it was expected according to the AUE description, algorithms based on AUE achieve better results than methods where the AWE is the base ensemble approach. This is due to, in the case of AWE, the use of a limited number of samples for each member, which impairs their discriminatory ability. Classifiers in AUE-based ensembles generally receive more samples from the same concept and thus better recognize the patterns they represent. For a similar reason, in the case of high imbalance models using *oversampling* cope better with the problem. It is related to the size of the received chunk, and more specifically to the number of received minority class objects. For the stream with the highest disparity between classes, each chunk contains only 25 samples of the minority class. After conducting *undersampling*, individual classifiers use very few samples to train, which results in their lower quality.

**Figure 1:**
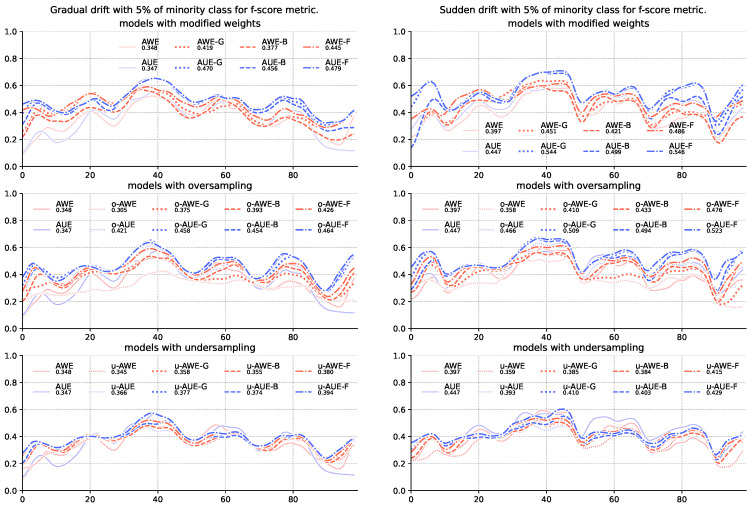
Comparison of base algorithms and their modifications, showing average F1-score value for each chunk of the stream with gradual and sudden concept drifts and 5% of minority class samples.

The obtained results show that changes in the weighting method have the greatest impact in the case of the F1-score metric (Table 3). What is more, introducing data sampling degrades the quality of ensembles using imbalanced metrics to calculate weights of member classifiers (Figure 1). Sampling directly affects the frequency of pointing to the minority class, which, by increasing the number of correctly recognized samples, also increases the number of samples falsely identified as a positive class-indicated with the precision metric used by the F1-score. Especially at high imbalance levels, when there are very few minority class samples, even a small percentage of poorly recognized majority class samples rapidly reduces the value of the precision metric. This also explains the significant difference between the values of the F1-score metric and the G-mean and balanced accuracy score in the case of the highest imbalance streams. The latter uses the specificity instead of precision, which, due to the large size of the majority class, responds much more mildly to incorrect classification of individual samples.

**Figure 2:**
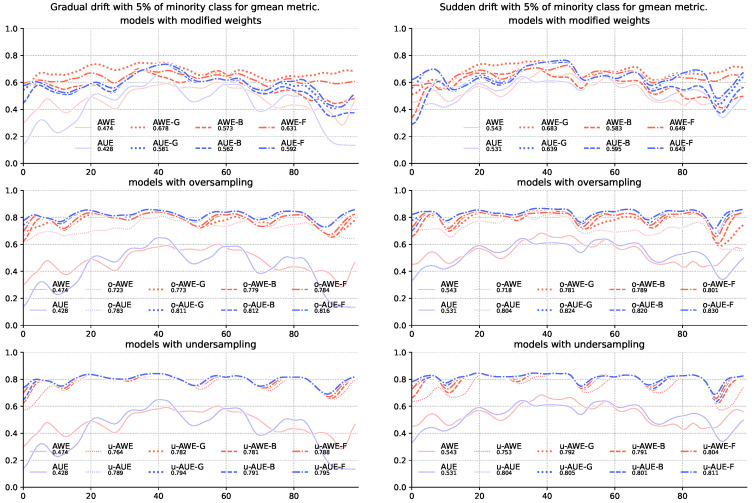
Comparison of base algorithms and their modifications, showing average G-mean value for each chunk of the stream with gradual and sudden concept drifts and 5% of minority class samples.

**Figure 3:**
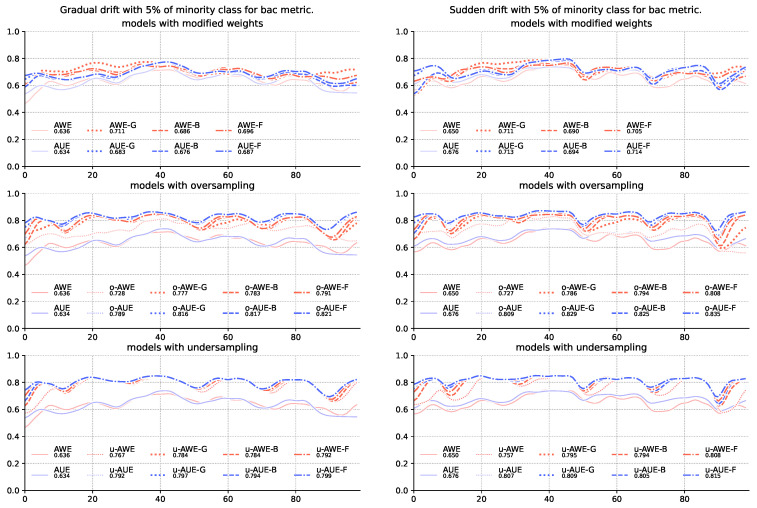
Comparison of base algorithms and their modifications, showing average balanced accuracy score for each chunk of the stream with gradual and sudden concept drifts and 5% of minority class samples.

The results for the G-mean (Table 4) and balanced accuracy score (Table 5) metrics show that the mere modification of the method of assigning weights to team members is insufficient—models using sampling alone were statistically significantly better than models without sampling. Both *under*-and *oversampling* significantly increased the quality of recognition of majority class objects with a slight deterioration in the classification of the majority class. Still, however, the addition of modification of weight allocation increases the quality of classification, which in some cases is also supported by statistical tests (Figure 2 and Figure 3).

**Figure 4:**
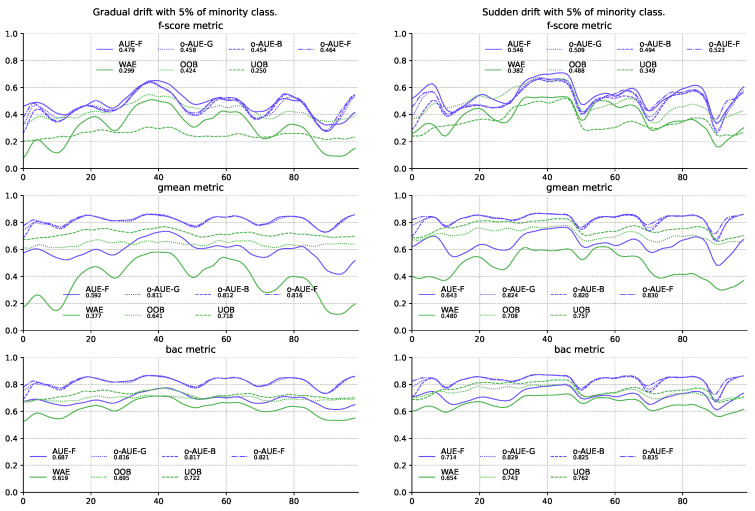
Comparison of the best proposed models with other methods of stream processing using the Test-Then-Train procedure on the stream with gradual and sudden concept drifts and 5% of minority class samples.

According to the results, the best method to assign weights seems to be in proportion to the F1-score and the second is in proportion to the G-mean metric. Both methods of calculating weights improve the quality of classifiers not only in relation to the own metrics used, but also in all the others. In addition, models using them are in most cases much better than almost all others, which also finds confirmation in performed statistical tests (Figure 4).

It is also worth noting that the proposed models with modifications are also suitable for problems with low imbalance and achieve much better quality than models created strictly for the problem of imbalanced data streams.

## 5. Conclusions

This paper presents a novel proposition extending state-of-the-art streaming data processing methods with modified weighting metrics for member-classifiers, taking into account the prior probability of classes present during the flow of data stream containing various types of concept drift phenomenon. An in-depth experimental analysis of the proposed methods was carried out, including three standard aggregated metrics used to assess the quality prediction models constructed on imbalanced classification problems, as well as statistical testing to verify the significance of differences between models. Experiments were conducted using various types of class imbalance and drift types to thoroughly study the characteristics of evaluated algorithms. In comparison with the standard methods of solving the problem of imbalanced data streams, based on the resampling of the training set, greater usefulness potential of the presented proposal has been demonstrated in all types of examined imbalance levels and occurring concept drifts. Nonetheless, the considerable limitation of this study was the lack of evaluation on real-life data, which should be included in further research, together with the additional introduction of proposed modifications to different stream processing algorithms.

The modifications introduced in the AWE and AUE methods allow a noticeable improvement in the predictive capabilities of ensemble models both in cases of high imbalance and with relatively small disproportions between the problem classes. The proposed method only modifies the method of establishing weights for individual classifiers in the ensemble pool, and therefore does not create any additional computational overhead, so without major contraindications it may be recommended to use in solving problems of imbalanced stream classification with any imbalance ratio. 

## Figures and Tables

**Table 1:** Comparison of data streams processed during experimental evaluation of modified models, consisting of the type of occurring concept drifts, information on what percent of all samples belong to minority class and the ratio between samples from both classes.

#	DRIFT TYPE	MINORITY CLASS %	CLASS RATIO
1	sudden	5%	1:19
2	sudden	10%	1:9
3	sudden	20%	1:4
4	sudden	30%	3:7
5	gradual	5%	1:19
6	gradual	10%	1:9
7	gradual	20%	1:4
8	gradual	30%	3:7

**Table 2:** Description of proposed models, including base ensemble algorithms, implemented changes—the way weights are calculated and used data sampling—and labels shown on plots.

#	BASE ENSEMBLE	WEIGHTING METHOD	SAMPLING	PLOT LABEL
1	AWE	proportional to *G-mean*	undersampling	u-AWE-g
2		proportional to *balanced accuracy score*	undersampling	u-AWE-b
3		proportional to *F1-score*	undersampling	u-AWE-f
4		proportional to *G-mean*	oversampling	o-AWE-g
5		proportional to *balanced accuracy score*	oversampling	o-AWE-b
6		proportional to *F1-score*	oversampling	o-AWE-f
7		proportional to *G-mean*	—	AWE-g
8		proportional to *balanced accuracy score*	—	AWE-b
9		proportional to *F1-score*	—	AWE-f
10		in inverse proportion to MSE	undersampling	u-AWE
11		in inverse proportion to MSE	oversampling	o-AWE
12	AUE	proportional to *G-mean*	undersampling	u-AUE-g
13		proportional to *balanced accuracy score*	undersampling	u-AUE-b
14		proportional to *F1-score*	undersampling	u-AUE-f
15		proportional to *G-mean*	oversampling	o-AUE-g
16		proportional to *balanced accuracy score*	oversampling	o-AUE-b
17		proportional to *F1-score*	oversampling	o-AUE-f
18		proportional to *G-mean*	—	AUE-g
19		proportional to *balanced accuracy score*	—	AUE-b
20		proportional to *F1-score*	—	AUE-f
21		in inverse proportion to MSE	undersampling	u-AUE
22		in inverse proportion to MSE	oversampling	o-AUE

**Table 3:** Average value of the F1-score metric for all compared models and every data stream type, with subscript containing a list of other methods, that are statistically worse for the given stream type.

#	METHOD	SUDDEN DRIFT				GRADUAL DRIFT			
		5%	10%	20%	30%	5%	10%	20%	30%
1	AWE	0.385	0.496	0.690	0.780	0.358	0.495	0.674	0.760
	${}^{undersampling+gmean}$	${}^{-}$	${}^{-}$	${}^{27}$	${}^{26:27}$	${}^{27}$	${}^{27}$	${}^{27}$	${}^{26:27}$
2	AWE	0.384	0.486	0.704	0.781	0.355	0.483	0.681	0.758
	${}^{undersampling+bac}$	${}^{-}$	${}^{-}$	${}^{26:27}$	${}^{26:27}$	${}^{27}$	${}^{27}$	${}^{27}$	${}^{26:27}$
3	AWE	0.415	0.515	0.722	0.785	0.380	0.505	0.690	0.761
	${}^{undersampling+fscore}$	${}^{-}$	${}^{10,27}$	${}^{26:27}$	${}^{26:27}$	${}^{11,27}$	${}^{27}$	${}^{27}$	${}^{26:27}$
4	AWE	0.410	0.547	0.720	0.783	0.375	0.507	0.690	0.761
	${}^{oversampling+gmean}$	${}^{-}$	${}^{10,27}$	${}^{26:27}$	${}^{26:27}$	${}^{27}$	${}^{27}$	${}^{27}$	${}^{26:27}$
5	AWE	0.433	0.577	0.720	0.784	0.393	0.539	0.688	0.763
	${}^{oversampling+bac}$	${}^{-}$	${}^{1:2,10,27}$	${}^{26:27}$	${}^{26:27}$	${}^{11,27}$	${}^{11,27}$	${}^{27}$	${}^{26:27}$
6	AWE	0.476	0.612	0.734	0.791	0.426	0.567	0.699	0.767
	${}^{oversampling+fscore}$	${}^{1:2,10,22,27}$	${}^{1:3,10:11,27}$	${}^{10,26:27}$	${}^{26:27}$	${}^{11,27}$	${}^{10:11,27}$	${}^{27}$	${}^{26:27}$
7	AWE	0.451	0.579	0.722	0.784	0.419	0.538	0.681	0.755
	${}^{gmean}$	${}^{-}$	${}^{10,27}$	${}^{26:27}$	${}^{26:27}$	${}^{11,27}$	${}^{27}$	${}^{27}$	${}^{26:27}$
8	AWE	0.421	0.600	0.725	0.785	0.377	0.548	0.686	0.756
	${}^{bac}$	${}^{-}$	${}^{1:2,10:11,27}$	${}^{26:27}$	${}^{26:27}$	${}^{-}$	${}^{27}$	${}^{27}$	${}^{26:27}$
9	AWE	0.486	0.627	0.742	0.791	0.445	0.569	0.692	0.760
	${}^{fscore}$	${}^{-}$	${}^{1:3,10:11,27}$	${}^{10,26:27}$	${}^{26:27}$	${}^{11,27}$	${}^{10:11,27}$	${}^{27}$	${}^{26:27}$
10	AWE	0.359	0.429	0.628	0.740	0.345	0.449	0.624	0.744
	${}^{undersampling}$	${}^{-}$	${}^{-}$	${}^{-}$	${}^{-}$	${}^{27}$	${}^{-}$	${}^{-}$	${}^{27}$
11	AWE	0.358	0.464	0.663	0.741	0.305	0.442	0.646	0.741
	${}^{oversampling}$	${}^{-}$	${}^{-}$	${}^{-}$	${}^{-}$	${}^{-}$	${}^{-}$	${}^{-}$	${}^{27}$
12	AWE	0.397	0.550	0.674	0.744	0.348	0.518	0.679	0.763
		${}^{-}$	${}^{27}$	${}^{-}$	${}^{-}$	${}^{-}$	${}^{27}$	${}^{27}$	${}^{26:27}$
13	AUE	0.410	0.582	0.740	0.810	0.377	0.548	0.707	0.787
	${}^{undersampling+gmean}$	${}^{-}$	${}^{2,10,27}$	${}^{10,26:27}$	${}^{11:12,26:27}$	${}^{11,27}$	${}^{10:11,27}$	${}^{26:27}$	${}^{26:27}$
14	AUE	0.403	0.567	0.733	0.807	0.374	0.541	0.708	0.786
	${}^{undersampling+bac}$	${}^{-}$	${}^{10,27}$	${}^{26:27}$	${}^{26:27}$	${}^{27}$	${}^{10:11,27}$	${}^{26:27}$	${}^{26:27}$
15	AUE	0.429	0.598	0.750	0.818	0.394	0.557	0.714	0.791
	${}^{undersampling+fscore}$	${}^{-}$	${}^{1:2,10:11,27}$	${}^{10,26:27}$	${}^{10:12,26:27}$	${}^{11,27}$	${}^{10:11,27}$	${}^{26:27}$	${}^{26:27}$
16	AUE	0.509	0.657	0.776	0.828	0.458	0.604	0.741	0.805
	${}^{oversampling+gmean}$	${}^{1:2,10:11,14,22,27}$	${}^{1:3,10:11,22,26:27}$	${}^{1:2,10:12,26:27}$	${}^{10:12,26:27}$	${}^{1:2,10:11,22,27}$	${}^{1:3,10:11,27}$	${}^{10:11,26:27}$	${}^{10:11,26:27}$
17	AUE	0.494	0.645	0.756	0.819	0.454	0.607	0.737	0.803
	${}^{oversampling+bac}$	${}^{1:2,10:11,22,27}$	${}^{1:3,10:11,22,26:27}$	${}^{10:12,26:27}$	${}^{10:12,26:27}$	${}^{1:2,10:11,27}$	${}^{1:3,10:11,27}$	${}^{10:11,26:27}$	${}^{10:11,26:27}$
18	AUE	0.523	0.663	0.779	0.831	0.464	0.610	0.743	0.806
	${}^{oversampling+fscore}$	${}^{1:3,10:11,13:14,22,27}$	${}^{1:4,10:11,22,26:27}$	${}^{1:2,10:12,26:27}$	${}^{10:12,26:27}$	${}^{1:2,10:11,22,25,27}$	${}^{1:3,10:11,27}$	${}^{10:11,26:27}$	${}^{10:11,26:27}$
19	AUE	0.544	0.671	0.775	0.821	0.470	0.613	0.735	0.796
	${}^{gmean}$	${}^{1:4,10:11,13:14,22,27}$	${}^{1:5,10:12,14,22,26:27}$	${}^{1:2,10:12,26:27}$	${}^{10:12,26:27}$	${}^{11,27}$	${}^{1:3,10:11,27}$	${}^{10,26:27}$	${}^{26:27}$
20	AUE	0.499	0.646	0.757	0.815	0.456	0.611	0.732	0.794
	${}^{bac}$	${}^{-}$	${}^{1:3,10:11,22,26:27}$	${}^{10:12,26:27}$	${}^{10:12,26:27}$	${}^{11,27}$	${}^{1:3,10:11,27}$	${}^{10,26:27}$	${}^{26:27}$
21	AUE	0.546	0.682	0.780	0.827	0.479	0.618	0.740	0.797
	${}^{fscore}$	${}^{1:4,10:11,13:14,22,27}$	${}^{1:5,10:14,22,26:27}$	${}^{1:2,10:12,26:27}$	${}^{10:12,26:27}$	${}^{10:11,27}$	${}^{1:3,10:11,27}$	${}^{10:11,26:27}$	${}^{26:27}$
22	AUE	0.393	0.543	0.746	0.813	0.366	0.522	0.707	0.788
	${}^{undersampling}$	${}^{-}$	${}^{10,27}$	${}^{10,26:27}$	${}^{10:12,26:27}$	${}^{11,27}$	${}^{27}$	${}^{27}$	${}^{26:27}$
23	AUE	0.467	0.610	0.760	0.820	0.421	0.563	0.724	0.800
	${}^{oversampling}$	${}^{10}$	${}^{1:3,10:11,27}$	${}^{10:12,26:27}$	${}^{10:12,26:27}$	${}^{11,27}$	${}^{10:11,27}$	${}^{10,26:27}$	${}^{26:27}$
24	AUE	0.447	0.642	0.766	0.820	0.347	0.547	0.736	0.798
		${}^{-}$	${}^{1:3,10:11,26:27}$	${}^{1,10:12,26:27}$	${}^{10:12,26:27}$	${}^{-}$	${}^{27}$	${}^{10:11,26:27}$	${}^{26:27}$
25	WAE	0.382	0.571	0.745	0.805	0.299	0.460	0.698	0.774
		${}^{-}$	${}^{-}$	${}^{10,26:27}$	${}^{26:27}$	${}^{-}$	${}^{-}$	${}^{27}$	${}^{26:27}$
26	OOB	0.488	0.529	0.624	0.679	0.424	0.524	0.624	0.682
		${}^{-}$	${}^{10,27}$	${}^{-}$	${}^{-}$	${}^{11,27}$	${}^{27}$	${}^{-}$	${}^{-}$
27	UOB	0.349	0.440	0.605	0.682	0.250	0.412	0.581	0.678
		${}^{-}$	${}^{-}$	${}^{-}$	${}^{-}$	${}^{-}$	${}^{-}$	${}^{-}$	${}^{-}$

**Table 4:** Average value of the G-mean metric for all compared models and every data stream type, with subscript containing a list of other methods, that are statistically worse for the given stream type.

#	METHOD	SUDDEN DRIFT				GRADUAL DRIFT			
		5%	10%	20%	30%	5%	10%	20%	30%
1	AWE	0.792	0.791	0.826	0.845	0.781	0.804	0.822	0.832
	${}^{undersampling+gmean}$	${}^{7:9,11:12,19:21,24:26}$	${}^{10:12,26}$	${}^{12,26:27}$	${}^{26:27}$	${}^{7:9,11:12,19:21,24:27}$	${}^{7:12,19:21,24:27}$	${}^{26:27}$	${}^{26:27}$
2	AWE	0.791	0.771	0.836	0.845	0.781	0.777	0.826	0.831
	${}^{undersampling+bac}$	${}^{7:9,11:12,19:21,24:26}$	${}^{10,12,26}$	${}^{10,12,26:27}$	${}^{26:27}$	${}^{7:9,11:12,19:21,24:27}$	${}^{11:12,24:26}$	${}^{12,26:27}$	${}^{26:27}$
3	AWE	0.804	0.780	0.844	0.848	0.788	0.789	0.828	0.833
	${}^{undersampling+fscore}$	${}^{7:12,19:21,24:26}$	${}^{10,12,26}$	${}^{10,12,26:27}$	${}^{12,26:27}$	${}^{7:9,11:12,19:21,24:27}$	${}^{8,10:12,19:21,24:27}$	${}^{12,26:27}$	${}^{26:27}$
4	AWE	0.781	0.799	0.842	0.846	0.773	0.785	0.827	0.833
	${}^{oversampling+gmean}$	${}^{7:9,12,19:21,24:25}$	${}^{10:12,20,26}$	${}^{10,12,26:27}$	${}^{26:27}$	${}^{7:9,11:12,19:21,24:27}$	${}^{11:12,24:26}$	${}^{12,26:27}$	${}^{26:27}$
5	AWE	0.789	0.819	0.842	0.847	0.779	0.810	0.826	0.834
	${}^{oversampling+bac}$	${}^{7:9,11:12,19:21,24:26}$	${}^{2,7:12,19:21,24:27}$	${}^{10,12,26:27}$	${}^{26:27}$	${}^{7:9,11:12,19:21,24:27}$	${}^{7:12,19:21,24:27}$	${}^{12,26:27}$	${}^{26:27}$
6	AWE	0.801	0.831	0.847	0.851	0.784	0.815	0.830	0.836
	${}^{oversampling+fscore}$	${}^{7:12,19:21,24:26}$	${}^{1:3,7:12,19:21,24:27}$	${}^{10:12,26:27}$	${}^{12,26:27}$	${}^{7:9,11:12,19:21,24:27}$	${}^{7:12,19:21,24:27}$	${}^{10,12,26:27}$	${}^{26:27}$
7	AWE	0.683	0.733	0.807	0.836	0.678	0.725	0.786	0.817
	${}^{gmean}$	${}^{25}$	${}^{-}$	${}^{26}$	${}^{26:27}$	${}^{12,24:25}$	${}^{-}$	${}^{-}$	${}^{26:27}$
8	AWE	0.583	0.729	0.809	0.837	0.573	0.712	0.786	0.818
	${}^{bac}$	${}^{-}$	${}^{-}$	${}^{26}$	${}^{26:27}$	${}^{-}$	${}^{-}$	${}^{-}$	${}^{26:27}$
9	AWE	0.649	0.735	0.815	0.840	0.631	0.714	0.786	0.820
	${}^{fscore}$	${}^{-}$	${}^{-}$	${}^{26}$	${}^{26:27}$	${}^{24:25}$	${}^{-}$	${}^{-}$	${}^{26:27}$
10	AWE	0.753	0.704	0.761	0.804	0.764	0.744	0.769	0.815
	${}^{undersampling}$	${}^{8:9,12,19:21,24:25}$	${}^{-}$	${}^{-}$	${}^{-}$	${}^{7:9,11:12,19:21,24:26}$	${}^{11:12,25}$	${}^{-}$	${}^{26:27}$
11	AWE	0.718	0.707	0.783	0.805	0.723	0.709	0.780	0.810
	${}^{oversampling}$	${}^{12,24:25}$	${}^{-}$	${}^{-}$	${}^{-}$	${}^{12,19:21,24:26}$	${}^{-}$	${}^{-}$	${}^{-}$
12	AWE	0.543	0.681	0.762	0.798	0.474	0.647	0.769	0.819
		${}^{-}$	${}^{-}$	${}^{-}$	${}^{-}$	${}^{-}$	${}^{-}$	${}^{-}$	${}^{26:27}$
13	AUE	0.805	0.832	0.858	0.868	0.794	0.822	0.843	0.853
	${}^{undersampling+gmean}$	${}^{7:12,19:21,24:26}$	${}^{1:3,7:12,19:21,24:27}$	${}^{7:12,26:27}$	${}^{10:12,26:27}$	${}^{7:9,11:12,19:21,24:27}$	${}^{2:3,7:12,19:21,24:27}$	${}^{7:12,25:27}$	${}^{26:27}$
14	AUE	0.801	0.824	0.852	0.865	0.791	0.819	0.843	0.853
	${}^{undersampling+bac}$	${}^{7:9,11:12,19:21,24:26}$	${}^{2,7:12,19:21,24:27}$	${}^{7,10:12,26:27}$	${}^{10:12,26:27}$	${}^{7:9,11:12,19:21,24:27}$	${}^{2:3,7:12,19:21,24:27}$	${}^{7:12,25:27}$	${}^{26:27}$
15	AUE	0.811	0.840	0.863	0.874	0.795	0.823	0.845	0.856
	${}^{undersampling+fscore}$	${}^{7:12,19:21,24:27}$	${}^{1:3,7:12,19:21,24:27}$	${}^{7:12,20,25:27}$	${}^{7:8,10:12,26:27}$	${}^{7:9,11:12,19:21,24:27}$	${}^{2:3,7:12,19:21,24:27}$	${}^{7:12,25:27}$	${}^{11,26:27}$
16	AUE	0.824	0.859	0.881	0.881	0.811	0.844	0.865	0.867
	${}^{oversampling+gmean}$	${}^{1:2,4:12,19:21,24:27}$	${}^{1:5,7:12,19:22,24:27}$	${}^{1:12,19:21,24:27}$	${}^{1:5,7:12,26:27}$	${}^{4:5,7:12,19:21,24:27}$	${}^{2:4,7:12,19:21,24:27}$	${}^{1,7:12,19:21,24:27}$	${}^{7:12,26:27}$
17	AUE	0.820	0.853	0.866	0.874	0.812	0.846	0.862	0.866
	${}^{oversampling+bac}$	${}^{1,4:5,7:12,19:21,24:27}$	${}^{1:5,7:12,19:22,24:27}$	${}^{7:12,20,25:27}$	${}^{7:8,10:12,26:27}$	${}^{1,4:5,7:12,19:21,24:27}$	${}^{2:4,7:12,19:21,24:27}$	${}^{7:12,19:21,24:27}$	${}^{7:12,26:27}$
18	AUE	0.830	0.866	0.882	0.883	0.816	0.849	0.866	0.868
	${}^{oversampling+fscore}$	${}^{1:2,4:12,19:21,23:27}$	${}^{1:14,19:22,24:27}$	${}^{1:12,19:21,24:27}$	${}^{1:5,7:12,25:27}$	${}^{1:2,4:12,19:21,23:27}$	${}^{1:5,7:12,19:21,24:27}$	${}^{1,7:12,19:21,24:27}$	${}^{7:12,26:27}$
19	AUE	0.639	0.749	0.837	0.863	0.581	0.712	0.810	0.847
	${}^{gmean}$	${}^{-}$	${}^{-}$	${}^{10,12,26:27}$	${}^{10:12,26:27}$	${}^{-}$	${}^{-}$	${}^{26:27}$	${}^{26:27}$
20	AUE	0.595	0.733	0.824	0.858	0.562	0.708	0.808	0.845
	${}^{bac}$	${}^{-}$	${}^{-}$	${}^{12,26:27}$	${}^{11:12,26:27}$	${}^{-}$	${}^{-}$	${}^{26:27}$	${}^{26:27}$
21	AUE	0.643	0.756	0.839	0.868	0.592	0.713	0.813	0.848
	${}^{fscore}$	${}^{-}$	${}^{26}$	${}^{10,12,26:27}$	${}^{10:12,26:27}$	${}^{25}$	${}^{-}$	${}^{26:27}$	${}^{26:27}$
22	AUE	0.804	0.814	0.859	0.868	0.789	0.813	0.840	0.854
	${}^{undersampling}$	${}^{7:12,19:21,24:26}$	${}^{7:12,19:21,24:27}$	${}^{7:12,25:27}$	${}^{10:12,26:27}$	${}^{7:9,11:12,19:21,24:27}$	${}^{7:12,19:21,24:27}$	${}^{10:12,26:27}$	${}^{26:27}$
23	AUE	0.804	0.841	0.869	0.875	0.783	0.822	0.852	0.863
	${}^{oversampling}$	${}^{7:12,19:21,24:26}$	${}^{1:3,7:12,19:21,24:27}$	${}^{7:12,20,25:27}$	${}^{10:12,26:27}$	${}^{7:9,11:12,19:21,24:27}$	${}^{2,7:12,19:21,24:27}$	${}^{7:12,25:27}$	${}^{7:8,10:12,26:27}$
24	AUE	0.531	0.726	0.830	0.862	0.428	0.632	0.808	0.847
		${}^{-}$	${}^{-}$	${}^{12,26:27}$	${}^{10:12,26:27}$	${}^{-}$	${}^{-}$	${}^{26}$	${}^{26:27}$
25	WAE	0.480	0.669	0.815	0.852	0.377	0.547	0.781	0.830
		${}^{-}$	${}^{-}$	${}^{26}$	${}^{12,26:27}$	${}^{-}$	${}^{-}$	${}^{-}$	${}^{26:27}$
26	OOB	0.708	0.686	0.735	0.754	0.641	0.706	0.745	0.759
		${}^{12,24:25}$	${}^{-}$	${}^{-}$	${}^{-}$	${}^{12,24:25}$	${}^{-}$	${}^{-}$	${}^{-}$
27	UOB	0.757	0.757	0.776	0.774	0.718	0.744	0.763	0.772
		${}^{8:9,12,19:21,24:25}$	${}^{12,26}$	${}^{-}$	${}^{-}$	${}^{12,19:21,24:26}$	${}^{11:12,25}$	${}^{-}$	${}^{-}$

**Table 5:** Average value of the balanced accuracy score metric for all compared models and every data stream type, with subscript containing a list of other methods, that are statistically worse for the given stream type.

#	METHOD	SUDDEN DRIFT				GRADUAL DRIFT			
		5%	10%	20%	30%	5%	10%	20%	30%
1	AWE	0.795	0.796	0.827	0.846	0.784	0.806	0.823	0.833
	${}^{undersampling+gmean}$	${}^{7:9,12,19:21,24:25}$	${}^{10:12,26}$	${}^{26:27}$	${}^{26:27}$	${}^{7:9,11:12,19:21,24:27}$	${}^{10:12,24:27}$	${}^{26:27}$	${}^{26:27}$
2	AWE	0.794	0.791	0.837	0.846	0.784	0.794	0.827	0.832
	${}^{undersampling+bac}$	${}^{7:9,12,19:21,24:25}$	${}^{10:12,26}$	${}^{10,12,26:27}$	${}^{26:27}$	${}^{7:9,11:12,19:21,24:27}$	${}^{10:12,24:27}$	${}^{26:27}$	${}^{26:27}$
3	AWE	0.807	0.805	0.846	0.849	0.791	0.803	0.830	0.834
	${}^{undersampling+fscore}$	${}^{7:12,19:21,24:26}$	${}^{10:12,26:27}$	${}^{10,12,26:27}$	${}^{26:27}$	${}^{7:9,11:12,19:21,24:27}$	${}^{8,10:12,20,24:27}$	${}^{26:27}$	${}^{26:27}$
4	AWE	0.785	0.801	0.843	0.848	0.777	0.787	0.829	0.834
	${}^{oversampling+gmean}$	${}^{7:9,12,19:21,24:25}$	${}^{10:12,26}$	${}^{10,12,26:27}$	${}^{26:27}$	${}^{7:9,11:12,19:21,24:27}$	${}^{11:12,25}$	${}^{26:27}$	${}^{26:27}$
5	AWE	0.794	0.821	0.843	0.848	0.783	0.812	0.827	0.835
	${}^{oversampling+bac}$	${}^{7:9,12,19:21,24:25}$	${}^{7:8,10:12,20,24:27}$	${}^{10,12,26:27}$	${}^{26:27}$	${}^{7:9,11:12,19:21,24:27}$	${}^{7:8,10:12,20,24:27}$	${}^{26:27}$	${}^{26:27}$
6	AWE	0.807	0.834	0.849	0.852	0.791	0.818	0.832	0.838
	${}^{oversampling+fscore}$	${}^{7:12,19:21,24:26}$	${}^{1:2,7:12,19:21,24:27}$	${}^{10:12,26:27}$	${}^{12,26:27}$	${}^{7:9,11:12,19:21,24:27}$	${}^{7:12,19:21,24:27}$	${}^{10,26:27}$	${}^{26:27}$
7	AWE	0.711	0.753	0.816	0.840	0.711	0.744	0.795	0.822
	${}^{gmean}$	${}^{-}$	${}^{-}$	${}^{26}$	${}^{26:27}$	${}^{25}$	${}^{-}$	${}^{-}$	${}^{26:27}$
8	AWE	0.690	0.758	0.818	0.840	0.686	0.744	0.797	0.822
	${}^{bac}$	${}^{-}$	${}^{-}$	${}^{26}$	${}^{26:27}$	${}^{-}$	${}^{-}$	${}^{-}$	${}^{26:27}$
9	AWE	0.705	0.765	0.825	0.844	0.696	0.747	0.799	0.825
	${}^{fscore}$	${}^{-}$	${}^{-}$	${}^{26:27}$	${}^{26:27}$	${}^{-}$	${}^{-}$	${}^{-}$	${}^{26:27}$
10	AWE	0.757	0.710	0.762	0.806	0.767	0.750	0.771	0.816
	${}^{undersampling}$	${}^{12,25}$	${}^{-}$	${}^{-}$	${}^{-}$	${}^{9,11:12,19:21,24:26}$	${}^{11}$	${}^{-}$	${}^{27}$
11	AWE	0.727	0.712	0.785	0.806	0.728	0.714	0.782	0.812
	${}^{oversampling}$	${}^{-}$	${}^{-}$	${}^{-}$	${}^{-}$	${}^{12,24:26}$	${}^{-}$	${}^{-}$	${}^{-}$
12	AWE	0.650	0.712	0.771	0.802	0.636	0.706	0.786	0.825
		${}^{-}$	${}^{-}$	${}^{-}$	${}^{-}$	${}^{-}$	${}^{-}$	${}^{-}$	${}^{26:27}$
13	AUE	0.809	0.835	0.859	0.869	0.797	0.825	0.844	0.855
	${}^{undersampling+gmean}$	${}^{7:12,19:21,24:26}$	${}^{2,7:12,19:21,24:27}$	${}^{7,10:12,26:27}$	${}^{10:12,26:27}$	${}^{7:9,11:12,19:21,24:27}$	${}^{2,7:12,19:21,24:27}$	${}^{7,10:12,26:27}$	${}^{26:27}$
14	AUE	0.805	0.828	0.853	0.866	0.794	0.822	0.844	0.854
	${}^{undersampling+bac}$	${}^{7:9,11:12,19:21,24:25}$	${}^{7:12,20,24:27}$	${}^{10:12,26:27}$	${}^{10:12,26:27}$	${}^{7:9,11:12,19:21,24:27}$	${}^{2,7:12,19:21,24:27}$	${}^{10:12,26:27}$	${}^{26:27}$
15	AUE	0.815	0.843	0.864	0.875	0.799	0.826	0.846	0.857
	${}^{undersampling+fscore}$	${}^{7:12,19:21,24:27}$	${}^{1:3,7:12,19:21,24:27}$	${}^{7:12,26:27}$	${}^{10:12,26:27}$	${}^{7:9,11:12,19:21,24:27}$	${}^{2,7:12,19:21,24:27}$	${}^{7:12,26:27}$	${}^{11,26:27}$
16	AUE	0.829	0.861	0.882	0.882	0.816	0.846	0.866	0.868
	${}^{oversampling+gmean}$	${}^{1:2,4:5,7:12,19:21,24:27}$	${}^{1:5,7:12,19:22,24:27}$	${}^{1:12,19:21,24:27}$	${}^{1:5,7:12,26:27}$	${}^{1,4:5,7:12,19:21,24:27}$	${}^{2:4,7:12,19:21,24:27}$	${}^{1,7:12,19:20,24:27}$	${}^{7:12,26:27}$
17	AUE	0.825	0.855	0.867	0.875	0.817	0.848	0.863	0.867
	${}^{oversampling+bac}$	${}^{1:2,4:5,7:12,19:21,24:27}$	${}^{1:5,7:12,19:22,24:27}$	${}^{1,7:12,25:27}$	${}^{7,10:12,26:27}$	${}^{1:2,4:5,7:12,19:21,24:27}$	${}^{2:4,7:12,19:21,24:27}$	${}^{7:12,20,25:27}$	${}^{7:12,26:27}$
18	AUE	0.835	0.868	0.883	0.884	0.821	0.851	0.867	0.869
	${}^{oversampling+fscore}$	${}^{1:12,19:21,24:27}$	${}^{1:14,19:22,24:27}$	${}^{1:12,19:20,24:27}$	${}^{1:5,7:12,26:27}$	${}^{1:2,4:5,7:12,19:21,24:27}$	${}^{1:5,7:12,19:21,24:27}$	${}^{1:2,5,7:12,19:20,24:27}$	${}^{7:12,26:27}$
19	AUE	0.713	0.781	0.846	0.867	0.683	0.756	0.824	0.851
	${}^{gmean}$	${}^{-}$	${}^{10,26}$	${}^{10,12,26:27}$	${}^{10:12,26:27}$	${}^{-}$	${}^{-}$	${}^{26:27}$	${}^{26:27}$
20	AUE	0.694	0.768	0.833	0.862	0.676	0.754	0.822	0.850
	${}^{bac}$	${}^{-}$	${}^{-}$	${}^{10,12,26:27}$	${}^{10:12,26:27}$	${}^{-}$	${}^{-}$	${}^{26:27}$	${}^{26:27}$
21	AUE	0.714	0.787	0.849	0.872	0.687	0.757	0.826	0.853
	${}^{fscore}$	${}^{-}$	${}^{10:12,26}$	${}^{10:12,26:27}$	${}^{10:12,26:27}$	${}^{-}$	${}^{-}$	${}^{26:27}$	${}^{26:27}$
22	AUE	0.807	0.819	0.861	0.870	0.792	0.816	0.841	0.855
	${}^{undersampling}$	${}^{7:12,19:21,24:26}$	${}^{7:8,10:12,20,25:27}$	${}^{7:8,10:12,26:27}$	${}^{10:12,26:27}$	${}^{7:9,11:12,19:21,24:27}$	${}^{7:12,19:21,24:27}$	${}^{10:12,26:27}$	${}^{26:27}$
23	AUE	0.809	0.843	0.870	0.876	0.789	0.824	0.853	0.864
	${}^{oversampling}$	${}^{7:12,19:21,24:26}$	${}^{1:3,7:12,19:21,24:27}$	${}^{1,7:12,25:27}$	${}^{10:12,26:27}$	${}^{7:9,11:12,19:21,24:27}$	${}^{7:12,19:21,24:27}$	${}^{7:12,26:27}$	${}^{7:8,10:12,26:27}$
24	AUE	0.676	0.766	0.839	0.865	0.634	0.726	0.824	0.853
		${}^{-}$	${}^{-}$	${}^{10,12,26:27}$	${}^{10:12,26:27}$	${}^{-}$	${}^{-}$	${}^{26:27}$	${}^{26:27}$
25	WAE	0.654	0.739	0.826	0.855	0.620	0.691	0.802	0.836
		${}^{-}$	${}^{-}$	${}^{26:27}$	${}^{11:12,26:27}$	${}^{-}$	${}^{-}$	${}^{-}$	${}^{26:27}$
26	OOB	0.743	0.724	0.755	0.766	0.695	0.735	0.760	0.768
		${}^{12}$	${}^{-}$	${}^{-}$	${}^{-}$	${}^{25}$	${}^{-}$	${}^{-}$	${}^{-}$
27	UOB	0.761	0.759	0.778	0.776	0.722	0.747	0.765	0.773
		${}^{12,24:25}$	${}^{26}$	${}^{-}$	${}^{-}$	${}^{12,24:25}$	${}^{-}$	${}^{-}$	${}^{-}$
